# Racism and antiracism in nursing education: confronting the problem of whiteness

**DOI:** 10.1186/s12912-022-00929-8

**Published:** 2022-06-10

**Authors:** Sharissa Hantke, Verna St. Denis, Holly Graham

**Affiliations:** University of Saskatchewan, Saskatoon, SK Canada

**Keywords:** Antiracism, Whiteness, Nursing education, Racism, Critical race theory

## Abstract

**Background:**

Systemic racism in Canadian healthcare may be observed through racially inequitable outcomes, particularly for Indigenous people. Nursing approaches intending to respond to racism often focus on culture without critically addressing the roots of racist inequity directly. In contrast, the critical race theory approach used in this study identifies whiteness as the underlying problem; a system of racial hierarchy that accords value to white people while it devalues everyone else.

**Methods:**

This qualitative study seeks to add depth to the understanding of how whiteness gets performed by nursing faculty and poses antiracism education as a necessary tool in addressing the systemic racism within Canadian healthcare. The methodology of poststructural discourse analysis is used to explore the research question: how do white nursing faculty draw on common discourses to produce themselves following introductory antiracism education?

**Results:**

Analysis of data reveals common patterns of innocent and superior white identity constructions including benevolence, neutrality, Knowing, and exceptionalism. While these patterns are established in other academic fields, the approaches and results of this study are not yet common in nursing literature.

**Conclusions:**

The findings highlight the need for antiracism education at personal and policy levels beginning in nursing programs.

## Background

### Racist health outcomes

The publicly funded Canadian healthcare system could be considered a point of national pride distinguishing us from the United States of America (USA) [1]. However, the outcomes of this system demonstrate that equal care and equal health are not made accessible for everyone. Indigenous people are frequently provided with substandard care, [2, 3], and are subjected to disproportionate rates of poverty and illnesses including tuberculosis, human immunodeficiency virus (HIV), and diabetes [4–6]. The horrific death of Joyce Echaquan at the hands of Quebec nurses after they taunted her with anti-Indigenous stereotypes [3] has caused more nurses, managers, and nurse educators to recognize the widespread harms of racism within Canadian healthcare and the dire need for action. Despite national narratives of Canada as an accepting, diverse, multicultural country, our history demonstrates that from the early policies shaping the health of Canadians the healthcare system was created for, and continues to be for, the benefit of the white settler population and the simultaneous exclusion of Indigenous people, citizens of Colour, and immigrants of Colour [7]. A lack of public understanding of policies enacting racial exclusion enables white settler Canadians, including healthcare professionals, to participate in racist systems without seeing or understanding the racialized harms.

The racist violence of our system and its resulting harm, such as the numerous instances made evident in the *In Plain Sight* report [3], ought to be at the forefront of efforts to improve Canadian healthcare.

### Why not focus on culture?

Nursing approaches attempting to respond to systemic racism in healthcare frequently focus on cultural learning, as in increasing one’s knowledge about ethnic minority cultures [8, 9]. Healthcare could learn from what antiracist education scholar St. Denis [10] describes: “We started out a few decades ago in Aboriginal education believing that we could address the effects of racialization and colonization by affirming and validating the cultural traditions and heritage of Aboriginal peoples. There is increasing evidence that those efforts have limitations” (p. 178). Focusing on cultural education misdiagnoses the underlying problem, puts the onus for change onto the oppressed group, and absolves white people of responsibility for making changes [10]. Culturalist approaches within nursing reify discourses which *other* oppressed groups while either ignoring or inadequately focusing on race and racism [8]. Instead of focusing on learning *about* the Other, nursing needs anti-oppressive approaches which are critical of othering and approaches that seek to change students and society [11].

Sometimes cultural safety and cultural competence get conflated, as Curtis et al. [9] identify. While cultural competence more narrowly focuses on cross-cultural behaviours and acquiring knowledge about ethnic minority groups, cultural safety critically considers biases, stereotypes, power, and colonialism [9]. Bell [8] notes that “cultural safety and cultural humility pedagogies employ a more critical lens than previous iterations of culturally based approaches” (p. 3) such as cultural competence, transculturalism, and cultural sensitivity. Furthermore, Bell [8] argues that “cultural safety will not be possible to attain without explicit deconstruction of the white supremacist ideology that people in colonial and post-colonial states are socialized into so that people fundamentally understand and become accountable for their (our) oppressive and/or privileged behaviour” (p. 4).

To create an outcome of cultural safety for racially oppressed groups, we must address the racism underlying the harm. To address racism in the healthcare system, all healthcare workers, educators, and decision makers need the tools of antiracism education grounded in critical race theory and critical whiteness studies. We must work on understanding the attitudes and priorities of the healthcare system and how they enact harm so that instead of focusing on cultural learning as a solution to racist harm, we work to understand and address the underlying problem at the core of racialized health outcomes: whiteness.

### Relevant nursing literature

Since Vaughan’s 1997 article [12] asks if there really is racism in nursing and answers with a definitive “yes”, very little nursing literature has explored racism in nursing, and less still has named whiteness as underlying racism. Prior to 2020, searching for nursing literature that critically addresses racism revealed few results. Exceptions include: Blanchet Garneau, Browne, and Varcoe [13] highlighting the need for antiracist pedagogy in nursing; Hilario, Browne, and McFadden [14] identify democratic racism in nursing—discourses that attempt to justify contradictions between Canadian values of tolerance and equity, and Canadian racism; Tang and Browne’s [15] study identify various ways that racist stereotypes impact Indigenous patients’ access to care; Scammell and Olumide [16] describe white nurses as “unwittingly” perpetuating racism; Van Herk, Smith, and Andrew [17] urgently suggest that intersectionality and critical pedagogy become part of nursing education and practice; and Walker’s dissertation [18] demonstrates the need for cultural safety training and delineating important differences between cultural competence and cultural safety.

### How whiteness underpins racism

This study uses the term “whiteness” to point to the system of racial hierarchy that positions white racial identity at the top and thereby affords disproportionate power and privilege to people racialized as white at the expense of everyone not racialized as white [19]. The theoretical bases underpinning this research are Critical Race Theory (CRT) and Critical Whiteness Studies (CWS). Three particularly relevant shared tenets of these fields include (1) understanding racism as systemic and often invisible to white people [20]; (2) race and whiteness as socially constructed to serve white interests [21–23] as opposed to primarily biologically or genetically [24]; and (3) the necessity of directing the critical gaze away from those subjected to racism and toward those who are unduly privileged by racial dominance [20, 25]. As such, this study understands racial categories as having been constructed in the eighteenth century to serve an exploitative agenda which still causes widespread health inequity [26]. Although the concept of races as biologically distinct categories is outdated and disproven, underlying biases based on these debunked ideas still get reified in science and health fields [8, 27]. This study understands race to be immensely impactful because of its social construction and maintenance and therefore aims to keep a critical focus on how race and racism get produced.

Instead of recognizing racism as a primarily interpersonal phenomenon, this study urges readers to consider how systemic racism is constructed and maintained systemically through discourses. Of particular interest are discourses which function to position whiteness as superior and as innocent. Through identifying and analyzing the discursive resources [28] utilized by white settler nursing faculty, this study aims to highlight the need for personal antiracism learning for individual white nursing faculty, the need for inclusion of antiracism curriculum into nursing programs, and the need for an antiracism lens at the policy level of nursing programs.

Although this study examines Canadian healthcare and Canadian data, the findings may have relevance more broadly. The Canadian context has been shaped by colonization, and the resulting racial dynamics may have parallels wherever colonialism and European imperialism oppress Indigenous peoples with a “huge legacy of suffering and destruction” (p. 20) [29].

## Methods

### Researcher context and reflexivity

This research and the antiracism education preceding it were undertaken as part of Sharissa Hantke’s master’s thesis. Sharissa is a white settler cisgender woman and worked under the supervision of Indigenous scholar and antiracism education expert Dr. Verna St. Denis and Indigenous scholar, psychologist, and nursing faculty Dr. Holly Graham. Since Sharissa is a white settler critically studying how white nursing faculty perform whiteness, guidance and mentorship from Indigenous scholars was critical in mitigating the risks of perpetuating settler colonialism.

### Study design

This study seeks to add depth to understanding how whiteness gets performed by nursing faculty and thereby to examine how the performance of whiteness functions to uphold racism within nursing education. The research question is therefore: how do white nursing faculty draw on common discourses to produce themselves following introductory antiracism education?

Following approval from the behavioural research ethics board, nursing faculty were recruited to a focus group interview to discuss their experience of a workshop introducing antiracism education. Such education is new to Saskatchewan nursing faculty, and the workshop sessions were made possible through funding from the Indigenous Research Chair of Canadian Institute of Health Research through the Dr. Graham’s wahkohtowin project. Although the entire workshop was 6 full days long, the training was offered in three two-day chunks, with four month breaks between. Due to time constraints, the 1.5 h focus group session was held after the first two-day workshop where participants had learned about race as a social construct, intersectionality, racism as systemic, history of racism in Canadian health care, debunking meritocracy, and exploring resistance to antiracist education. Recruitment occurred at the end of day two of the workshop session by inviting white nursing faculty to participate in the research study. Of the 24 registrants who met eligibility requirements for being white nursing faculty, three volunteered to participate in the focus group, and informed consent was obtained. These three white nursing faculty teach in different nursing programs, and their voluntary participation demonstrates their keenness to continue learning antiracism. These participants were provided with open ended reflection questions in advance which asked them about difficult/uncomfortable parts of the education, their anticipation of how the education may impact their teaching, and their next steps on their antiracism journey. The focus group interview was semi structured to allow for participants to respond to each other and build off each other’s responses in the group context. One strength of a focus group approach was that the conversation allowed participants to continue to learn from and to build relationships with each other. The focus group was facilitated by the primary author. These participants were at different points in their antiracism learning and this is reflected in the data which came from all three participants.

The focus group dialogue was transcribed and then discourses were identified consistent with critical race theory (CRT) literature [30]. Concept mapping was used to track discourses which connect to CRT. Data was interpreted from the poststructural discourse analysis perspective of language as not simply a means of neutrally describing reality—rather of discourses as doing particular things [28].

Wetherell [28] says that identities are “constituted as they are formulated in discourse” (p. 12). Focus group participants used their words to produce their own white nursing faculty identities in patterns consistent with literature in the field of Critical Whiteness Studies [21, 31–34]. Critically examining how white people construct identity is imperative because of the connections to racialized health disparities.

Inequality is not first a fact of nature and then a topic of talk. Discourse is intimately involved in the construction and maintenance of inequality. Inequality is constructed and maintained when enough discursive resources can be mobilized to make colonial practices of land acquisition, for instance, legal, natural, normal, and ‘the way we do things.’ (p. 13) [28]

Therefore it is imperative to examine the sorts of discourses white nursing faculty employ so that we can learn how to identify deeply held beliefs which find their way into nursing practices and into policy, thereby producing racist outcomes (see Structural Determinism framework [35]). In seeking out these discourses, this research aims to gain more understanding of how white settlers contribute to racist harm and how to work toward both personal and policy level change.

As such, this study examines common, well-intentioned, and seemingly benign discursive maneuvers present in the focus group interview not for the purpose of critiquing the individuals who made the statements in the time they generously volunteered to the study, but rather to examine what these common statements and sentiments do in regards to race.

## Results

The data presented here reveal two aspects of whiteness so that white nurses and white nursing faculty can identify how whiteness functions. Those of us who inhabit whiteness find it difficult to identify [36] and thus whiteness must be “made strange” for white people to study it critically [37]. The seven pieces of data here each represents a commonplace example drawing upon discursive resources consistent with patterns of the performance of whiteness as established in CRT and CWS literature. Discursive maneuvers are presented here as they relate to two broad categories: innocence and superiority. Data were chosen for their commonplaceness; none of the discourses employed were unusual or surprising. Rather, they are oft-repeated, routine narratives [28] available to and employed by white settlers in Canada.

### Innocence

Tuck and Yang [38] identify that settler “moves to innocence” function to relieve guilt while abnegating responsibility for change. Employing discourses of innocence can redirect focus from the responsibility of addressing inequity to our own benevolence and good intentions. Innocence in this context can be understood as innocence from racism, where the definition of racism is limited to intentional interpersonal harm resulting from moral failing [32]. Scheurich and Young [39] point out that using this faulty definition of racism enables white university faculty to absolve themselves of personal responsibility. There is thus much to be accomplished in constructions of white innocence. The disadvantages racist policies and practices produce for Indigenous people in Canada relate directly to the advantages white settlers receive [40]. Rather than focus on Indigenous people as though they are the problem, this research highlights examples of discourses of innocence through which whiteness is produced.

This first piece of data is illustrative of innocent benevolence: “I just try to always remember that we ー I think we’re nice people and we’re coming from a good place and hopefully people understand that. That’s what I keep repeating to myself.” These sentences direct the focus onto the benevolent intentions of white people thereby precluding considering any harmful impact of one’s words or actions. The implication that nice white people must be innocent of racism serves to obscure the group members’ participation in racially unjust systems. Although the antiracism education may have destabilized white participants’ self-conception, rather than exploring a more complex identity such as “being an anti-racist racist” [41], the above statement insists on a nice, good, innocent understanding of self.

In addition to benevolence, white constructions of innocence may deploy neutrality through refusing to consider the racial power one holds. This second piece of data, a rhetorical strategy of neutrality is utilized to construct an innocent self. In reflecting upon what to do when someone makes a racist comment, one participant said, “you know sometimes even those comments, when people make those to you, do I [sic] stay neutral?” This sentence equates remaining silent as a sign of neutrality rather than seeing silence as a means of supporting or enabling the racist comment. Portraying the decision to be silent as somehow maintaining neutrality can function to grant the racist comment freedom to pass undisputed, or the silence can imply agreement. The phrasing of “staying neutral” reveals the speaker’s understanding of their white self as already neutral. Such rhetorical strategies of neutrality reinforce the construction of white innocence when white people speak as though our (Sharissa) whiteness does not position us with disproportionate power at the expense of Black people, Indigenous people, and people of Colour.

**Fig. 1:**
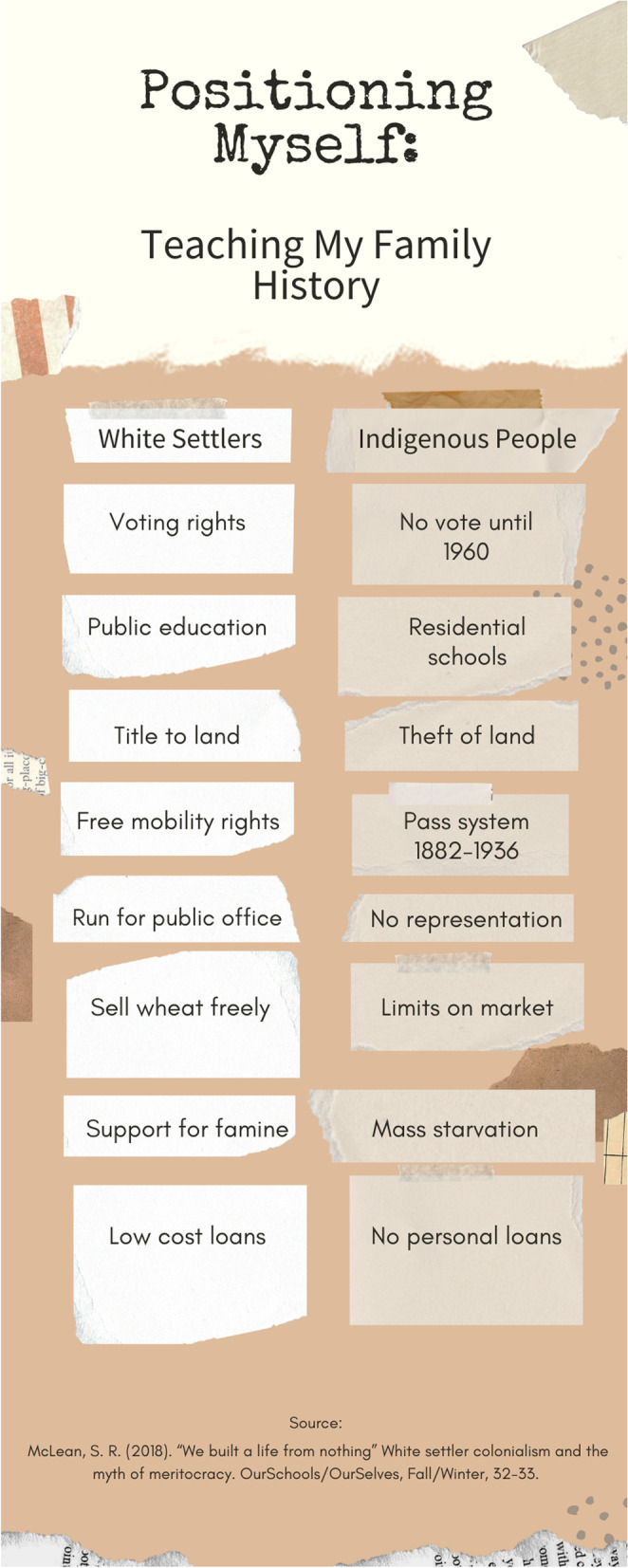
Positioning myself

In a third data sample participants were asked to consider their next steps. The participant intended to tell their students that “you have to put your biases aside and treat every- do your best to treat everyone the same.” This statement once again seeks to construct white people as neutral and separable from their biases, ignoring white people’s deep socialization into white dominance [42]. The idealized notion of treating everyone the same is an example of meritocratic colourblind discourse. The meritocratic aspect of the statement functions to uphold the myth that all people start from the same place and therefore success is based solely on hard work and determination, as portrayed in McLean’s [43] impactful article that dispels the myth of meritocracy. Such a myth overlooks historical racial inequity produced by policies demonstrated in Fig. 1. The colourblind aspect of the statement overlooks present day racial inequity by refusing to acknowledge difference [44]. This sentiment prioritizes sameness of treatment over the need to equitably address historical and present day racist harm. Through colourblind meritocratic discourses, the statement attempts to conceal sentiments which uphold the harmful status quo within seemingly open language.

### Superiority

At the core of the construct of white racial identity is a deeply ingrained sense of superiority [45]. This section considers how identifying as a Knower and as exceptional are examples of a broader pattern of understanding the white self as superior. Superiority is a relative term; for superiority to be possible implies the necessity of inferiority as comparison. There are many ways that a sense of superiority can manifest, and the two demonstrated here were selected for their relevance among well-intentioned, open-minded white people who do not want to be (seen as) racist. Positioning oneself as a Knower can have the impact of seeming superior through authoritativeness. To be the one who knows is valued over being one who does not know, and thus this construction is desired as superior. Exceptionalism here means portraying oneself as somehow special, one of the “good ones,” and elevated above “those other” racist whites. Exceptionalism and being the Knower can go hand in hand, particularly when white people construct ourselves as exceptional by means of our antiracist knowledge.

The fourth piece of data is brief: “We know more than we think.” This was stated as the group articulated their intended next steps in antiracism. Neither the veracity nor the intention of this sentence will be examined here. Instead, we look at what the statement *does*. In context, it was being used to comfort the group of white nursing faculty by validating their knowledge. It constructs the group as those who already know and it could be an effort to avoid the discomfort of *not knowing*. Both the discomfort experienced in antiracist, anti-oppressive education, and the crisis that arises in this process, are necessary components of this education and ought not to be avoided [46, 47].

Here is the fifth piece of data: “That’s what makes me angry, it makes me angry sometimes… the people that maybe really needed to be there don’t come to those things.” This statement refers to the participants’ colleagues who did not attend the antiracism education. By distinguishing the white focus group as those who showed up in contrast with those who actually needed the education, this statement positions the group members as exceptional. By creating a dichotomy of the racist ones who really need it and the good ones who showed up, the statement positions the workshop attendees and the keen focus group participants as not “really needing” antiracism education in contrast to those who did not join. Such positioning is at odds with participants’ descriptions of how much they learned during the sessions. How then, can they imply that they did not “really need” to be there? Additionally, emotions can be understood to *do things* [48] and we can understand the statement’s disclosure of anger to highlight and reinforce the white focus group participants’ sense of white exceptionality [49–51].

In a sixth data sample, the speaker constructs their self as both a Knower and as exceptional in their communications with students: “If I don’t have those [antiracist] conversations and show that they can happen and make mistakes with my students then they’re never going to have those conversations, right?” The statement demonstrates a prioritization of critical conversations, showing an understanding of how necessary antiracism work is. At the same time, the statement portrays the speaker as the one and only means by which their students would be exposed to antiracist conversations, therefore constructing their own exceptionalism as well as an identity as a lone white antiracist hero [52]. The statement also implies that the speaker is prepared and competent to have critical antiracist conversations, and is thus also constructing the speaker as an antiracist Knower.

In their 2005 article [53], Schick and St. Denis state, “this is the assumption of superiority that whiteness permits: what we have and who we are is what the world needs, whether it wants it or not” (p. 308). White nursing faculty, and nurses more generally must work to identify and uproot assumptions of superiority. Since these assumptions have been embedded from our (Sharissa) very early socialization into whiteness [42], identifying and unlearning them is an undertaking which will only be possible with deep and humble and ongoing reflection using the tools available in the scholarship of critical race theory and critical whiteness studies. One such tool white people can practice using is understanding our (Sharissa) complicity in systemic racism.

### Complicity

Having examined data which provides examples that function to construct white participants as innocent and superior, patterns which support the racist status quo, it is now time to consider one piece of data which does something different. When debriefing about feelings that arose during the antiracism education, one participant recognized an important moment, “For me I think it was just like oh my. I’m contributing to this.” This seventh data statement points to a moment of clarity which contrasts to the discourses of innocence and superiority mentioned above, and points toward a process of realizing complicity in the harmful systems which uphold the status quo. For white people who want to work toward a racially equitable future, it is necessary to not attempt to set oneself apart from “those racist whites” but instead to recognize the ongoing racist harm caused by participating in racist systems. Rather than focusing energy on defending individual goodness or innocence, white people need to be open to the deeply uncomfortable idea that despite intentions, they cause racist harm.

Reflecting upon one’s complicity in harmful systems is distinct from the emotions of guilt which may arise with this reflective work. Guilt might emerge in the process of learning about one’s personal complicity with whiteness, but guilt can stall out the antiracist efforts of white people, and is therefore a state to be worked through and learned from, not one to linger in. “While guilt is often a sign of a much-needed shift in consciousness, in itself it does nothing to motivate the responsibility necessary to actively dismantle entrenched systems of oppression” [54].

It is important that white people do not center their own feelings and self in antiracism work [49]. Learning about the complexities of the systems of racial oppression they participate in must be personal, but personal reflection is not the goal of antiracism work in and of itself. Antiracism work must seek to address racial injustice and work toward racial equity outcomes. As Kendi [55] emphasizes, unless policy change happens, antiracist change is not happening.

## Discussion

These data provide a snapshot of some discursive resources [28] which are available and utilized by highly educated, well-intentioned, open-minded white people. The analysis of the above statements demonstrates the use of poststructural discourse analysis by critically examining what each discourse accomplishes. Since beliefs and biases find their way into action, practices, and policies, they therefore cause harmful outcomes for oppressed groups [25, 35, 53]. The deeply socialized sense of superiority which shows up subtly (or not) in white people’s language has real world implications, resulting in the perpetuation of inequity and therefore if white people want to practice antiracism, they must work to identify and unlearn these deep beliefs.

It is necessary to excavate deeply embedded senses of superiority if white people want to address the crux of racial inequity. This deep and disruptive work will not be accomplished by cultural learning but through critical examination of unequal power and oppression through antiracist approaches.

Regarding the recommendations these findings point toward, it may be tempting to think that nursing programs could address the harms of whiteness by simply adding an antiracism course to our curriculum. While the addition of such content is desperately needed and will be an important step in equipping new generations of nurses with the tools of antiracism, it alone will not be enough. Nursing educators must first work to develop an antiracism lens through which they constantly teach and practice. We need major paradigm shifts, similar to the shifts to health promotion of the late twentieth century, to bring our programs into alignment with antiracist, anti-oppressive approaches. To prepare for such necessary policy changes, nursing program faculty, staff, and administration will need to be equipped with antiracist education. Therefore, this research urges as next steps the preparation of nursing faculty, staff, and administration with a solid grounding in antiracism education.

Specific changes and incremental steps are not provided in this article, and indeed require further efforts to determine. “Antiracist traditions provide us with useful critiques of existing situations, but tools developed to challenge racism will not always serve equally well to envision new racial possibilities. Critical tools are shaped to an important degree by the relations they are meant to disrupt” [49] (p. 21).

### Strengths and limitations

Although the discourses considered in this paper are consistent with CRT and CWS literature, this study does not claim to be generalizable due to the small size and limited duration of the focus group. A significant limitation was the timing of the focus group session. The focus group happening after the first two-day educational workshop rather than after the third and final two-day workshop means that the discourses documented here emerged when the participants had only been introduced to very initial antiracism concepts. An advantage of the timing was that their discussion informed which content to prioritize in the following workshop days.

A strength of this research is that using poststructural discourse analysis to understand racism and whiteness is not yet common practice in nursing literature. Providing an example of the application of this methodology may demonstrate a tool which could be quite useful for nurses pursuing antiracist approaches.

## Conclusion

The deeply rooted problem of racism in Canadian healthcare necessitates change at the foundational level. If we want to eliminate the racialized health outcomes evident in our system, we need to understand our colonial context which values whiteness. Since whiteness is a structure which devalues all who are not white, we must learn to identify how whiteness functions so that we can unlearn these ways and instead actively build and practice change which prioritizes equity. One aspect of identifying and unlearning whiteness is developing a capacity to identify the patterns of whiteness. Tools and theories that can inform and support this crucial work are available, particularly in the fields of CRT and CWS, and it is time for nursing to learn from them and integrate them into our education programs.

## Data Availability

The datasets analysed during the current study are available from the corresponding author on reasonable request.

## Abbreviations

CRT: Critical Race Theory
CWS: Critical Whiteness Studies
